# Nerelimomab Alleviates Capsaicin-Induced Acute Lung Injury by Inhibiting TNF Signaling and Apoptosis

**DOI:** 10.3390/ph17121694

**Published:** 2024-12-15

**Authors:** Lijuan Huang, Bing Du, Xiaohu Cui, Hanqing Zhao, Yanlin Feng, Ziying Xu, Jianhai Long, Jing Yuan, Fuping You

**Affiliations:** 1 School of Basic Medical Sciences, Peking University Health Science Center, Beijing 100191, China; huanglijuanlmm@sina.com; 2 Department of Bacteriology, Capital Institute of Pediatrics, Beijing 100020, China; 3 Department of Pulmonary and Critical Care Medicine, Beijing Tiantan Hospital, Capital Medicine University, Beijing 100070, China

**Keywords:** capsaicin, transcriptome, proteome, exogenously induced acute lung injury, treatment

## Abstract

**Background:** Capsaicin is commonly used as a flavoring and a riot control agent. However, long-term exposure or high doses can cause acute lung injury in military and police personnel. The mechanisms underlying capsaicin-induced pulmonary toxicity remain unclear. Therefore, this study investigated the molecular mechanisms involved in capsaicin-induced acute lung injury using C57BL/6N mice. **Methods:** Through both transcriptomic and proteomic analyses of mouse lung tissue, we identified the involvement of the TNF signaling pathway in capsaicin-mediated acute lung injury. Next, we explored the role of TNF signaling in the progression of acute lung injury to identify potential therapeutic targets. In a capsaicin-induced acute lung injury mouse model and A549 cells, we assessed the therapeutic potential of the TNF-α antibody Nerelimomab. Compared with the control group, TNF-α up-regulation was observed, which correlated with increased pathological changes and elevated IL-6 (*p* < 0.01) and IL-18 (*p* < 0.01) levels, both in vivo and in vitro. **Results:** Flow cytometry revealed that compared to the capsaicin group, Nerelimomab treatment reduced the number of capsaicin-induced apoptotic cells (*p* < 0.001) and was associated with an increased Bcl-2/Bax ratio (*p* < 0.01) and reduced cleaved caspase 3 expression (*p* < 0.001). Analysis of A549 cells treated with capsaicin and Nerelimomab corroborated these results. These findings confirm the involvement of the TNF signaling pathway in capsaicin-induced acute lung injury and the apoptosis of alveolar epithelial cells. **Conclusions:** In conclusion, capsaicin inhalation can cause acute lung injury, and targeting the TNF signaling pathway offers a promising therapeutic strategy. Nerelimomab demonstrates significant potential in alleviating acute lung injury by inhibiting inflammatory mediator release and diminishing apoptosis. Based on transcriptomic and proteomic analyses, this study highlights the crucial role of the TNF signaling pathway in capsaicin-induced acute lung injury and supports the therapeutic efficacy of Nerelimomab in reducing epithelial apoptosis.

## 1. Introduction

Tear gas, a non-lethal or low-lethality riot control agent, is primarily used to maintain social order, stop violent crimes, combat smuggling and drug trafficking, and counter terrorist activities [1]. Common police riot control agents include the tear agent acetophenone (CN), the sneezing agent Adams (DM), and the tear and sneezing agents SIAR (CR), Sies (CS), and oily pepper (OC), as well as the new generation of capsaicin, PavA. Capsaicin, a natural extract, gained popularity as a new type of riot control agent due to its low stimulation threshold, rapid onset and disappearance of symptoms, and relatively high safety profile [2,3]. Additionally, capsaicin can be released through various means, making it widely used in police riot control [2,4]. Despite being a natural product, the injurious effects of capsaicin are often overlooked. Currently, research on the morbidity and mortality associated with capsaicin exposure is limited both domestically and internationally. Therefore, it is important to understand the toxicological mechanism and harmful effects of capsaicin, as is strengthening research on medical protective measures to effectively improve the combat capability of military and police personnel and prevent large-scale poisoning events.

Capsaicin, primarily derived from pepper and other capsicum plants, was discovered, purified, and named in 1846 [5]. Despite being considered relatively safe, capsaicin can cause serious complications and even death in cases of poisoning [4,6]. The intense irritation caused by capsaicin can lead to symptoms such as tearing, coughing, sneezing, upper respiratory tract pain, and difficulty breathing [7]. In enclosed spaces, high doses and prolonged exposure to capsaicin can result in severe complications and fatalities, with multiple cases of capsaicin poisoning reported [8,9,10]. The toxicological mechanisms and injuries induced by capsaicin are often overlooked. Moreover, research on the incidence and mortality rates associated with capsaicin, both domestically and internationally, is limited. Therefore, developing a comprehensive understanding of the toxicological mechanisms and injury effects of capsaicin, alongside identifying novel protective strategies, is crucial for improving the operational capabilities of military and law enforcement personnel and preventing large-scale poisoning incidents.

Alveolar epithelial cells form the foundation of lung structure and function. To this end, alveolar epithelial cell apoptosis plays an important role in both physiological and pathological conditions [11]. Inhalation of toxic chemicals leads to direct contact of these agents with alveolar epithelial cells, leading to cellular damage [12,13]. In case of chemical-induced acute lung injury (ALI), apoptosis of alveolar epithelial cells can result in the severe destruction of lung tissue and impairment of respiratory function [14,15]. Therefore, recent studies suggested that targeting the apoptotic pathway of alveolar epithelial cells may alleviate the extent and severity of ALI.

Tumor necrosis factor-alpha (TNF-α) plays a significant role in the pathophysiology of ALI [11,14,16]. Notably, ALI is caused by various intrapulmonary and extrapulmonary pathogenic factors and is characterized by an acute inflammatory process involving inflammatory cell infiltration, damage to the alveolar epithelial and alveolar capillary membranes, increased vascular permeability, and interstitial/alveolar edema [17]. During ALI development, TNF-α regulates alveolar epithelial cell damage and repair processes by activating several molecular signaling pathways, including MAPK, NF-κB, AMPK/SIRT1, PI3K/Akt, Nrf2/HO-1, Fas/FasL, Wnt/β-catenin, and Notch [18,19,20,21,22,23]. Specifically, TNF-α promotes the activation and infiltration of inflammatory cells and increases alveolar capillary membrane permeability, leading to alveolar edema, and further infiltration of inflammatory cells [21]. Additionally, TNF-α can induce apoptosis of alveolar epithelial cells, disrupt the alveolar–capillary barrier, and exacerbate lung injury [13,16]. Therefore, treatment strategies targeting TNF-α and its associated signaling pathways may alleviate ALI symptoms and improve lung function and prognosis.

Nerelimomab is a humanized monoclonal antibody designed to target and neutralize tumor necrosis factor-α (TNF-α), a key cytokine that plays a crucial role in the inflammatory process [21]. It works by binding to TNF-α, preventing it from interacting with receptors on the cell surface, and ultimately blocking downstream inflammatory cascades. This mechanism of action is similar to that of other TNF inhibitors, such as infliximab, which demonstrated significant clinical efficacy in managing inflammatory diseases. The development of Nerelimomab began in the early 1990s when Celltech collaborated with Bayer to develop and commercialize it as part of a broader effort to create novel therapies targeting inflammatory cytokines [19]. Nerelimomab is a non-fucosylated anti-human TNF therapeutic bispecific antibody, classified as an IgG1 type. The Fc fragment of Nerelimomab (Figure S1) binds to FcγR of the TNF-α receptor, in turn interfering with the normal binding of TNF-α to its receptor, thereby reducing the TNF-α-mediated inflammatory response [15]. Nerelimomab is used in the treatment of ankylosing spondylitis. Additionally, as a TNF inhibitor and immunosuppressant, Nerelimomab’s investigational indications include septic shock, Crohn’s disease, and rheumatoid arthritis. However, despite showing positive results in early clinical trials, later trials for septic shock did not demonstrate a significant reduction in mortality compared to the control group [11].

Regulating apoptosis in alveolar epithelial cells is crucial in managing ALI. Therefore, understanding the specific mechanisms underlying this programmed cell death in capsaicin-induced ALI is essential for the development of novel treatments. This study utilized transcriptomic and proteomic analyses of mouse lung tissue to first identify the significant role that the TNF signaling pathway plays in capsaicin-induced ALI. Subsequently, we used Nerelimomab to inhibit TNF signaling pathway activation, with the aim of reducing apoptosis and alleviating capsaicin-induced ALI both in vivo and in vitro. The findings of this study provide theoretical support for in-depth understanding of the toxicological mechanism of capsaicin and provide academic insights for strengthening medical protective measures, which are crucial for improving the operational capabilities of military and police personnel and preventing large-scale capsaicin poisoning incidents. Considering Nerelimomab has not been approved for clinical treatment, future research should focus on assessing its bioavailability, pharmacokinetics, and safety of Nerelimomab. Further, more applications of Nerelimomab in clinical treatment should be explored in mice with different genders and ages.

## 2. Results

### 2.1. Capsaicin Induces ALI In Vivo

**Figure 1:**
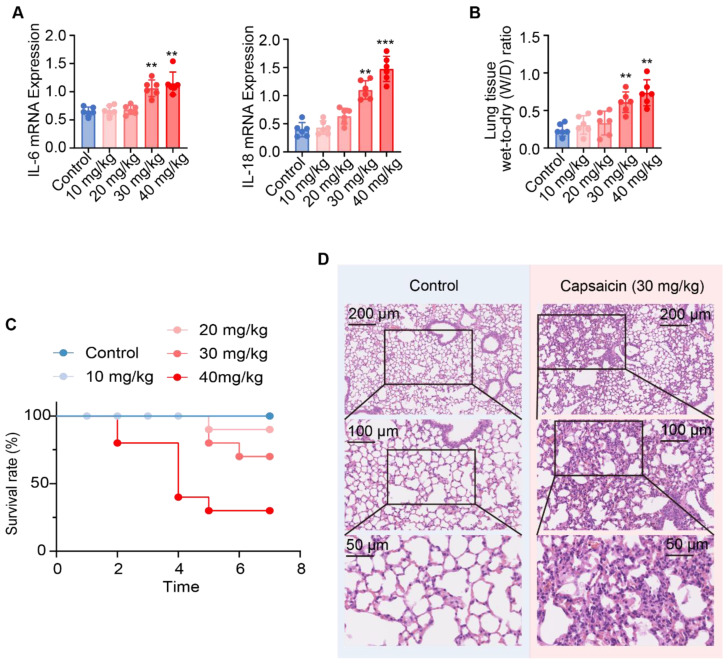
Capsaicin-induced acute lung injury. (**A**) IL-6 and IL-18 mRNA expression in C57BL/6N mice (n = 6); (**B**) change in lung tissue wet-to-dry (W/D) ratio (n = 6); (**C**) survival rates of C57BL/6N mice treated with capsaicin (n = 10); and (**D**) histopathological injury in the lungs of C57BL/6N mice treated with capsaicin (30 mg/kg). n = 6; data are represented as mean ± SD; ** *p <* 0.01 and *** *p* < 0.001 vs. the control group.

To assess whether capsaicin induces ALI, we measured IL-6 and IL-18 mRNA expression in lung tissue, the wet-to-dry (W/D) ratio of the lungs, and the survival rate of C57BL/6N mice, alongside conducting HE staining. After 3 days, zero of six mice treated with 10, 20, and 30 mg/kg capsaicin died; however, two of eight mice treated with 40 mg/kg capsaicin died. Notably, a significant upregulation of IL-6 mRNA expression was observed in 30 and 40 mg/kg capsaicin-treated groups compared to those in the control group (Figure 1A). Similarly, IL-18 mRNA levels were significantly elevated in the lung tissue of mice treated with 30 or 40 mg/kg capsaicin. Additionally, capsaicin treatment (30 and 40 mg/kg) led to a significant increase in the W/D ratio; this indicated that capsaicin treatment at these doses results in major inflammatory factor release in lung tissue (Figure 1B). Furthermore, the survival rates of C57BL/6N mice were found to be 70% and 25% 7 days after being treated with 30 and 40 mg/kg capsaicin, respectively (Figure 1C). Considering the IL-6 and IL-18 mRNA levels, W/D ratios, and survival rates, a dose of 30 mg/kg was selected for further in vivo experiments. In alignment, histopathological analysis of lung tissues from C57BL/6N mice treated with capsaicin (30 mg/kg) revealed moderate proliferation and thickening of alveolar septa, proliferation of lung epithelial cells, mild tissue parenchyma, atrophy of alveolar cavities, and diffuse infiltration of local inflammatory cells (Figure 1D).

### 2.2. Capsaicin-Induced Transcriptome and Proteome Changes

**Figure 2:**
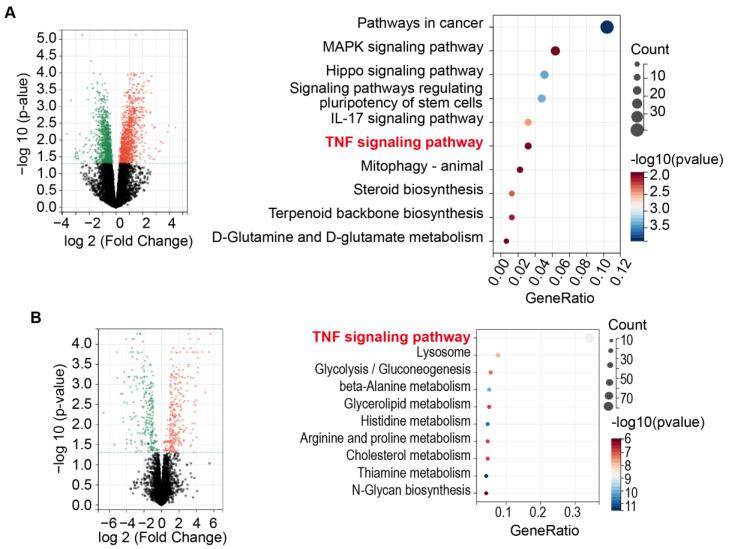
Omics analysis of lung tissue from C57BL/6N mice treated with capsaicin, (n = 3). (**A**) Transcriptome analysis; (**B**) Proteome analysis; n = 3.

To elucidate the toxicological mechanisms underlying capsaicin-induced ALI, we conducted transcriptome and proteome analyses of lung tissue from C57BL/6N mice treated with capsaicin. Differential expression and KEGG pathway enrichment analyses were performed using transcriptome data. Compared to the control group, the capsaicin-treated group possessed 374 down-regulated and 570 up-regulated genes (Figure 2A). KEGG pathway analysis revealed significant differences in several pathways; Figure 2A presents the top 10 pathways, with significant differences in the mRNA levels of genes associated with cancer pathways, MAPK signaling, Hippo signaling, regulation of stem cell pluripotency, IL-17 signaling, TNF signaling, animal mitophagy, steroid biosynthesis, terpenoid backbone biosynthesis, and D-glutamine and D-glutamate metabolism.

Subsequently, differential expression and enrichment analysis of the proteome data was performed. The findings demonstrate that compared to control group, the capsaicin-treated group possessed 367 down-regulated and 407 up-regulated proteins (Figure 2B). KEGG pathway analysis of differentially expressed proteins (DEPs) revealed significant differences in several pathways; Figure 2B displays the top 10 pathways with significant differences in protein expression levels, including TNF signaling, lysosome pathways, glycolysis/gluconeogenesis, beta-alanine metabolism, glycerolipid metabolism, histidine metabolism, arginine and proline metabolism, cholesterol metabolism, thiamine metabolism, and N-glycan biosynthesis. Notably, both transcriptome and proteome analysis indicated that the TNF signaling pathway plays a crucial role in capsaicin-induced ALI.

### 2.3. Nerelimomab Inhibits Apoptosis to Alleviate Capsaicin-Induced ALI In Vivo

**Figure 3:**
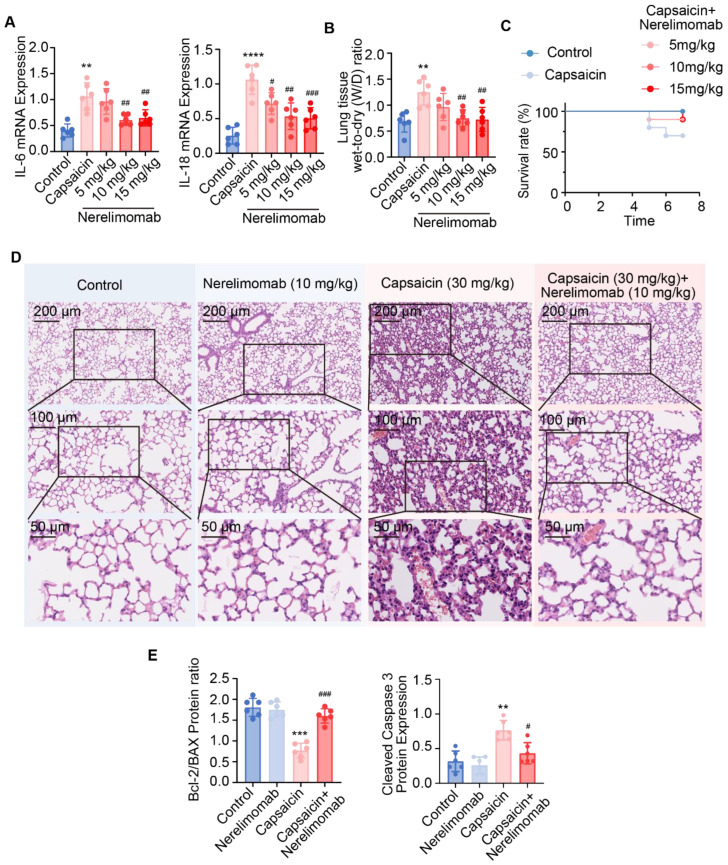
Nerelimomab alleviates capsaicin-induced acute lung injury by inhibiting apoptosis in vivo. (**A**) IL-6 and IL-18 mRNA expression in the lung tissue of C57BL/6N mice (n = 6); (**B**) change in lung tissue wet-to-dry (W/D) ratio (n = 6); (**C**) survival rates of C57BL/6N mice treated with capsaicin and Nerelimomab (n = 10); (**D**) histopathological injury in the lungs of mice with capsaicin-induced acute lung injury treated with Nerelimomab; and (**E**) Bcl-2/Bax protein ratio, and cleaved caspase 3 protein expression in the lung tissue of C57BL/6N mice (n = 6). Data are represented as mean ± SD; ** *p* < 0.01, *** *p* < 0.001, and **** *p* < 0.0001 vs. the control group. Data are represented as mean ± SD; ^#^ *p* < 0.05, ^##^ *p* < 0.01, and ^###^ *p* < 0.001 vs. the capsaicin group.

None (0 of 6) of the animals from each treatment group died. Notably, RT-qPCR analysis showed that IL-6 (*p <* 0.01) and IL-18 (*p <* 0.0001) mRNA expression levels were significantly increased in the capsaicin-treated group compared to controls (Figure 3A). Meanwhile, treatment with Nerelimomab at doses of 10 and 15 mg/kg significantly reduced IL-6 (*p <* 0.01) and IL-18 (*p <* 0.01 and *p <* 0.001) mRNA expression levels compared to those observed in the capsaicin group.

The lung tissue W/D ratio, an indicator of pneumonia [24,25], was significantly increased by capsaicin, but was reduced following Nerelimomab treatment (Figure 3C). Additionally, the survival rate of C57BL/6N mice improved from 70% to 90% after treatment with 10 mg/kg Nerelimomab (Figure 3C). Histopathological analysis further demonstrated that Nerelimomab treatment (10 mg/kg) ameliorated lung tissue injury induced by capsaicin (Figure 3D). Based on these findings, a dose of 10 mg/kg Nerelimomab was selected for subsequent experiments.

Finally, mRNA expression analysis revealed reduced TNF-α levels in the lung tissue of capsaicin-treated mice following administration of 10 mg/kg Nerelimomab (Figure S2). Moreover, apoptosis-related markers, including the Bcl-2/Bax ratio and cleaved caspase 3 protein expression, were assessed. Notably, capsaicin treatment significantly reduced the Bcl-2/Bax protein expression ratio (*p <* 0.001) and increased cleaved caspase 3 expression (*p <* 0.01), which is indicative of apoptosis. Meanwhile, the capsaicin + Nerelimomab group exhibited a significantly increased Bcl-2/Bax ratio (*p <* 0.01) and decreased cleaved caspase 3 expression (*p <* 0.05), suggesting that Nerelimomab inhibits apoptosis in capsaicin-induced ALI by modulating the TNF signaling pathway. The Bcl-2/Bax mRNA ratio and caspase 3 mRNA expression, shown in Figure S2, are consistent with the trend of protein level expression.

### 2.4. Nerelimomab Inhibits Alveolar Epithelial Cell Apoptosis via the TNF Signaling Pathway In Vitro

**Figure 4:**
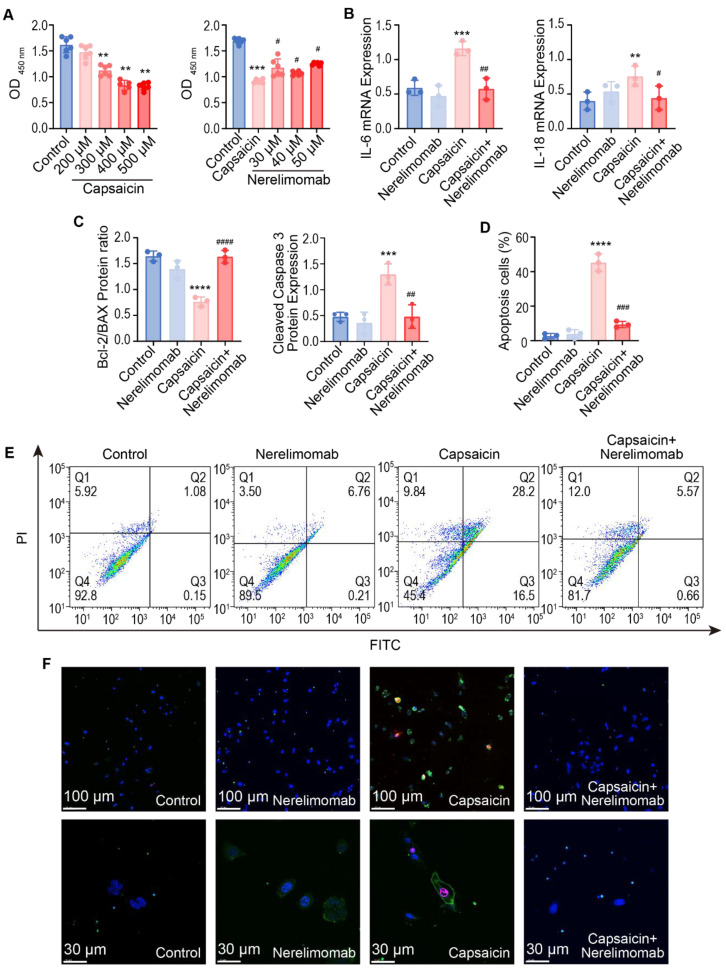
Nerelimomab alleviates capsaicin-induced alveolar epithelial cell injury by inhibiting apoptosis in vitro. (**A**) Change in A549 cell viability (n = 3); (**B**) mRNA expression of IL-6 and IL-18 (n = 3); (**C**) Bcl-2/Bax protein ratio, and cleaved caspase 3 protein expression (n = 3); (**D**) proportion of apoptotic A549 cells; (**E**) flow cytometry analysis of Annexin Ⅴ/PI staining for A549 cells apoptosis; and (**F**) confocal microscopy showing PI (red) and Annexin Ⅴ (green) staining of lung tissue sections. Results are representative of three independent experiments; data are presented as mean ± SD; ** *p <* 0.01, *** *p <* 0.001 and **** *p <* 0.0001 vs. the control group. Data are presented as mean ± SD; ^#^ *p* < 0.05, ^##^ *p <* 0.01, ^###^ *p* < 0.001 and ^####^ *p* < 0.0001 vs. the capsaicin group.

To further verify the role of the TNF signaling pathway in capsaicin-induced ALI, we conducted in vitro experiments using A549 alveolar epithelial cells treated with capsaicin and Nerelimomab. The CCK-8 assay indicated that capsaicin treatment (300 μM) significantly decreased A549 cells viability, whereas Nerelimomab treatment (30, 40, and 50 μM) significantly improved the viability of these cells (Figure 4A). Additionally, RT-qPCR analysis demonstrated that Nerelimomab treatment (40 and 50 μM) significantly decreased IL-6 and IL-18 expression levels that were increased by capsaicin treatment (300 μM). Based on these results, 300 μM capsaicin and 50 μM Nerelimomab were the selected doses used in subsequent experiments.

Notably, RT-qPCR analysis revealed that capsaicin significantly increased TNF-α expression in A549 cells (*p <* 0.0001), while Nerelimomab significantly decreased TNF-α expression (*p <* 0.01). To verify the role of TNF-α in epithelial cell apoptosis, we evaluated protein expression of apoptosis-related markers (Bcl-2/Bax ratio and caspase 3) in A549 cells by Elisa assay (Figure 4C). The Bcl-2/Bax mRNA ratio and caspase 3 mRNA expression, shown in Figure S3, are consistent with the trend of protein level expression. These results indicate that Nerelimomab effectively modulated these apoptosis-related markers, reducing capsaicin-induced apoptosis.

Flow cytometry analysis of Annexin V-FITC/PI-stained cells was used to identify the number of apoptotic cells in each treatment group (Q2 plus Q3). The compensation and gating strategy are shown in Figure S4. Notably, capsaicin treatment significantly increased the proportion of apoptotic cells compared to those in the control group (*p <* 0.0001) (Figure 3E and Figure 4D). However, the capsaicin + Nerelimomab group exhibited a significant reduction in the proportion of apoptotic cells compared to those in the capsaicin group (*p <* 0.001). Confocal microscopy also revealed differences in apoptotic cell morphology between these groups. In the capsaicin group, phosphatidylserine inversion, cell shrinkage, nuclear concentration, cell bubbling, and apoptotic body formation were observed and the Nerelimomab treatment led to significant improvements in these apoptosis-associated morphological changes (Figure 4F).

## 3. Discussion

Capsaicin is a natural product generally considered safe; however, this assumption may not be entirely accurate [2]. When used as a spray, capsaicin rapidly stimulates tear secretion, which can be accompanied by upper respiratory tract irritation symptoms, including nasal irritation, bronchoconstriction, severe coughing, sneezing, and shortness of breath [4]. Capsaicin exposure also causes skin burning sensations and neuromuscular dysfunction, such as motor control dysregulation [7]. While capsaicin-associated irritation is typically manageable, higher concentrations of capsaicin can cause severe respiratory and cardiovascular symptoms, along with permanent damage to the sensory nervous system [1]. As a result, several fatal cases of capsaicin exposure have been reported, most of which resulted in death within 1 h of exposure [6]. Notably, animal experiments have shown that the severity of capsaicin toxicity depends on the route of exposure, dosage, and age.

The acute reactions caused by capsaicin are primarily related to the respiratory system. Low concentrations of capsaicin induce coughing and sneezing, whereas high concentrations can cause bronchoconstriction, mucosal edema, dyspnea, and ALI; among these conditions, ALI is the leading cause of capsaicin-associated fatalities [1]. Specifically, capsaicin inhalation produces a strong tingling sensation in the nasal cavity and activates capsaicin-sensitive sensory nerves, resulting in vasodilation and increased secretion [26]. Earlier studies, including that by Maxwell and colleagues, found that inhaling 0.1 μmol/L capsaicin via nebulization led to an increase in inspiratory flow rate by 35 ± 6% compared with the placebo group in eight healthy volunteers (aged 25–35 years) [27]. One officer experienced immediate dyspnea upon exposure to capsaicin during a military training involving a tear-producing agent. Three months later, he developed persistent symptoms such as shortness of breath, coughing, wheezing, and dyspnea. Diagnosis through X-ray, chest CT, and lung function tests revealed that the severe cough triggered by capsaicin caused the rupture of subpleural pulmonary bullae, leading to mediastinal pneumatosis, pericardial pneumatosis, and mild pulmonary dysfunction [28]. Previous studies [29,30] suggest that capsaicin exerts its effects through a specific molecular receptor located on the terminals of primary afferent neurons in the brain and the cell membrane. This receptor is known as the capsaicin receptor (also called vanilloid receptor 1; VR1). Upon binding to VR1, capsaicin activates nonspecific cation channels, leading to the influx of cations (primarily Ca^2+^), which leads to membrane depolarization, action potential generation, and the transmission of nerve impulses along sensory fibers of the central nervous system, ultimately producing the sensation of pain. Furthermore, the influx of calcium ions, facilitated by VR1 receptors and voltage-dependent calcium channels, stimulates the release of neuropeptides and excitatory amino acids from nerve terminals. This cascade induces excitotoxic effects, defensive reflexes, and spontaneous movement. The acute biological responses elicited by capsaicin are associated with the release of bioactive substances from sensory nerve endings, such as substance P, neurokinin A, and calcitonin gene-related peptide. These substances alter the neurophysiology of sensory neurons in the respiratory mucosa, and induce neurogenic inflammation in respiratory epithelial cells, blood vessels, glands, and smooth muscle. Consequently, this results in bronchoconstriction, mucus secretion, tracheal mucosal edema, increased vascular permeability, apnea, and pulmonary edema.

However, the direct injury mechanism of capsaicin to lung tissue has not been reported. Specifically, further research into its toxicological mechanism and the development of effective first-aid prevention and treatment strategies is necessary.

To evaluate the effects and underlying mechanisms of capsaicin-induced respiratory tract injury, we employed a capsaicin-treated C57BL/6N mouse model. In vivo results indicate that mice treated with 30 mg/kg capsaicin for three days had significant upregulation of IL-6 and IL-18 mRNA levels in lung tissue. Additionally, a higher inhalation dose resulted in a more significant increase in IL-18 levels. The lung tissue W/D ratio serves as an indicator of inflammatory exudation. From the W/D ratio of lung tissue in mice, capsaicin inhalation at 30 or 40 mg/kg led to an imbalance in the generation and reflux of tissue fluid in the organs. This ultimately resulted in a large amount of tissue fluid extravasating from the pulmonary capillaries and accumulating in the alveoli, lung interstitium, and small bronchi, leading to pulmonary ventilation dysfunction. Moreover, survival data show that inhalation of 30 mg/kg capsaicin led to the death of 30% of the mice after seven days, while 40 mg/kg capsaicin led to a 75% mortality rate within the same timeframe.

Overall, our results demonstrate that inhalation of high doses of capsaicin leads to ALI, resulting in significant loss of lung function and increased mortality.

To investigate the toxicological mechanism of capsaicin-induced ALI, we analyzed the transcriptomes and proteomes of the lung tissues from mice exposed to capsaicin. Previous research indicates that the pathophysiology of ALI involves various mechanisms, including inflammatory cell infiltration, oxidative stress, alveolar capillary barrier disruption/permeability changes, apoptosis, and tissue fibrosis. In these processes, signaling pathways such as TNF-α and NF-κB are activated, and therefore, play crucial roles in the occurrence and development of ALI [11,21,31]. Consistent with these observations, our KEGG enrichment analysis of DEGs and DEPs indicated that the TNF signaling pathway is an important contributor to capsaicin-induced ALI.

ALI is a severe condition characterized by diffuse inflammation of the lung parenchyma and intractable hypoxemia. Various studies reported that upon stimulation, inflammatory cells, such as macrophages, neutrophils, and lymphocytes, release a large number of pro-inflammatory factors, including TNF-α [15,32]. These factors mediate the inflammatory response, leading to the accumulation and infiltration of inflammatory cells in and into lung tissue [31]. Subsequently, this process activates intracellular signal transduction pathways, leading to the release of additional inflammatory cytokines and further activation of additional inflammatory cells, thereby forming a vicious cycle that eventually leads to a cytokine storm [33].

**Figure 5:**
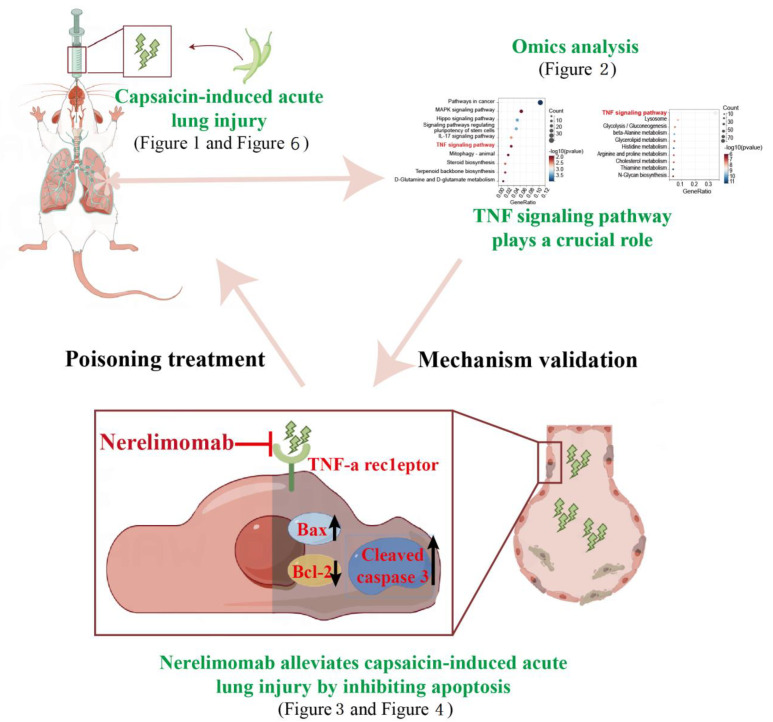
Nerelimomab alleviates capsaicin-induced acute lung injury by inhibiting the TNF signaling pathway and apoptosis in vivo and in vitro; (some of the material is from Figdraw https://www.figdraw.com/#/, accessed on 15 March 2023, and the authorization code is Pr=wqb2bb2).

TNF-α can also activate caspase cascades, leading to apoptosis, with abnormal apoptosis of alveolar epithelial cells being an important cause of ALI [19,20]. Given that capsaicin stimulates the release of many inflammatory factors (TNF-α) from lung tissue, we hypothesized that this abnormal activation of the TNF signaling pathway initiates the caspase cascade, leading to increased apoptosis of alveolar epithelial cells and the development of ALI. As shown in Figure 5, to confirm the role of the TNF signaling pathway in capsaicin-induced ALI, we used Nerelimomab, a mouse monoclonal antibody that acts as a TNF-α inhibitor. Our aim was to inhibit TNF signaling pathway activation, thereby reducing apoptosis and alleviating capsaicin-induced ALI both in vivo and in vitro. Nerelimomab treatment (10 and 15 mg/kg) reduced the mRNA expression of primarily pro-inflammatory cytokines (IL-6 and IL-18) and lung tissue W/D ratio, in turn improving the survival of C57BL/6N mice and alleviating capsaicin-induced pathological injury in lung tissue. Additionally, Nerelimomab treatment reduced TNF-α and cleaved caspase 3 mRNA expression and increased the Bcl-2/Bax mRNA ratio, indicating that Nerelimomab alleviates capsaicin-induced apoptosis.

A549 cells are alveolar type 2 epithelial cells commonly used in lung toxicity studies [34,35]. Therefore, we conducted a series of in vitro experiments using these cells and noted that capsaicin exposure reduced A549 cell viability, while Nerelimomab treatment improved it. Additionally, capsaicin-induced changes in TNF signaling pathway-related (IL-6, IL-18, and TNF-α) and apoptosis-related (Bcl-2/Bax and cleaved caspase 3) mRNA expressions were reversed by Nerelimomab treatment. These results corroborate our in vivo findings and indicate that capsaicin induces apoptosis of alveolar epithelial cells via the TNF signaling pathway.

TNF-α is a multifunctional cytokine that plays a central role in immune and inflammatory responses. It can lead to ALI or its more serious form, acute respiratory distress syndrome (ARDS), through a variety of mechanisms [2]. The main mechanisms of ALI induced by TNF-α include (1) TNF-α increasing the permeability of endothelial cells, making it easier for plasma proteins and inflammatory cells to cross the vascular wall into the alveolar space; (2) TNF-α activation of neutrophils and promotion of the release of proteases, oxygen free radicals, and other toxic substances, which can directly damage alveolar epithelial cells and endothelial cells; (3) TNF-α-induced production of other inflammatory mediators, such as interleukin-1β (IL-1β), interleukin-6 (IL-6), and prostaglandin E2 (PGE2), which further aggravate the inflammatory response; (4) TNF-α-induced apoptosis of alveolar epithelial cells and endothelial cells, and destruction of lung tissue integrity; and (5) TNF-α affecting the coagulation and fibrinolysis system, leading to microthrombosis and fibrin deposition [19]. These changes can damage the structure of pulmonary vessels and alveoli. In this study, significant changes in the expression of apoptosis-related molecules were found in animal lung tissues, and the changes in apoptosis-related indicators were reversed by an inhibitor. This study found that TNF-induced apoptosis plays an important role in capsaicin-induced ALI [36].

Capsaicin has been reported to be of use in the treatment of certain pain conditions by depleting the pain-transmitting substances of nerve endings through transient receptor potential vanilloid 1 (TRPV1) [37]. The TPRV1 mRNA expression is shown in Figure S5. The effect of capsaicin on inflammation is closely related to exposure concentration, time, as well as tissue and cell type. Low concentration capsaicin can lead to transient activation of the TRPV1 receptor, which may lead to desensitization or internalization of the receptor, reducing the reactivity of pain nerve endings to pain stimulation, thereby reducing inflammation-induced pain. A small amount of capsaicin inhibits inflammation by activating anti-inflammatory signaling pathways such as AMP-activated protein kinase (AMPK) [2]. High concentrations of capsaicin may lead to the continuous activation of the TRPV1 receptor, which does not cause desensitization, but results in the excessive excitement of nociceptive nerves and aggravation of the inflammatory response. Simultaneously, a large amount of capsaicin may promote the release of inflammatory mediators, such as increasing the synthesis of PGE2 and cytokines (such as TNF-α, IL-1β), thereby exacerbating inflammation [5]. The TRPV1 and TNF pathways interact and cross-act in cell signal transduction and the inflammatory response. Under inflammatory conditions, TNF-α increases the transcription and expression of TRPV1 by activating transcription factors such as nuclear factor-kappa B (NF-κB), thereby enhancing the inflammatory response of cells. TNF-α can activate the apoptosis pathway by binding to the TNF receptor. The activation of TRPV1 can also promote apoptosis by increasing intracellular calcium concentration and activating the caspase cascade [3]. However, in capsaicin-induced ALI, whether the effects are TRPV1-dependent or -independent and which cell types express TRPV1 receptors, remain to be investigated in future studies.

Despite the widespread availability of capsaicin in China for hundreds of years, our study based on in vitro and in vivo experiments showed that excessive consumption and respiratory inhalation can severely affect human health. Therefore, our study explored the effects of high-dose capsaicin on animal health, performing acute inhalation toxicity experiments in vitro and in vivo to provide a theoretical basis for the safe use of capsaicin. Future research should focus on assessing the bioavailability, pharmacokinetics and safety of Nerelimomab. Further, more applications of Nerelimomab in clinical treatment should be explored.

## 4. Materials and Methods

### 4.1. Chemicals

To verify the involvement of the TNF signaling pathway in capsaicin-induced ALI, we investigated the effects of Nerelimomab, a mouse monoclonal antibody, acting as a TNF-α antibody, on ALI and apoptosis. Capsaicin (≥95%) and Nerelimomab (≥95%) were purchased from MedChemExpress (Monmouth Junction, NJ, USA). Primary antibodies against actin, cleaved caspase 3, Bax, and Bcl-2 were purchased from Abcam Co., Ltd. (Birmingham, UK). The BCA Protein Quantitative Kit (KD22030122) was purchased from Kaiji Biotechnology Co., Ltd. (Nanjing, China). Meanwhile, Cell Counting Kit-8 (CCK-8) assay kits (CK04) were purchased from Dongren Chemical Technology Co., Ltd. (Shanghai, China).

### 4.2. Animals and Experimental Groups

Adult male C57BL/6N mice (8–10 weeks old, 20–22 g) were obtained from Vital River Laboratory Animal Technology Co., Ltd. (Beijing, China). All exposure experiments followed OECD test guidelines. All animal experiments were approved by the Medical Ethics Committee of the Capital Institute of Pediatrics (license number: DWLL2021009).

To determine the optimal capsaicin concentration for use in the animals, the concentration of capsaicin solution was adjusted according to the weight of each individual C57BL/6N mouse to ensure the quantity of capsaicin administered was 30 or 40 mg/kg, thereby creating two capsaicin treatment groups for this preliminary experiment. All treatments were administered via a nebulizer to ensure direct delivery of capsaicin to the lungs. Survival was monitored post-treatment by observing the mice for 7 days. Based on the survival curve and the changes in IL6 and IL18 in lung tissue, the concentrations of 10 and 30 mg/kg were selected for capsaicin and Nerelimomab, respectively, in the in vivo experiments.

**Figure 6:**
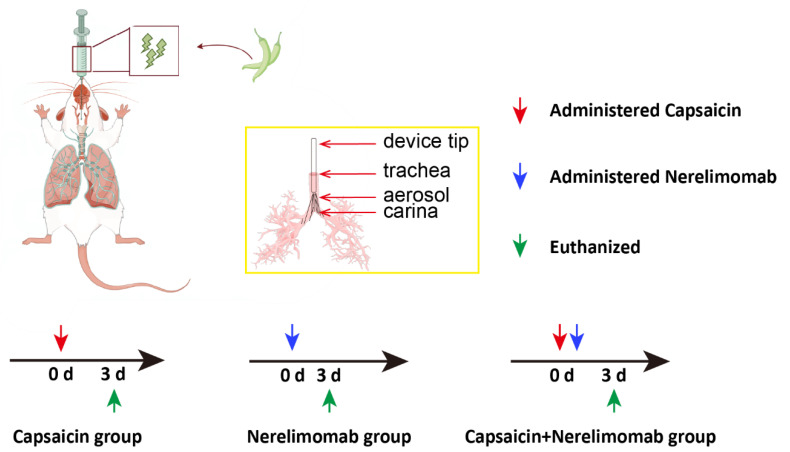
Treatment diagrams; (some of the material from Figdraw https://www.figdraw.com/#/, accessed on 15 March 2023, and the authorization code is Pr=wqb2bb2).

Subsequently, another set of mice was divided into four groups: saline control (50 μL, saline), Nerelimomab control (10 mg/kg), capsaicin-treated (30 mg/kg), and capsaicin (30 mg/kg) + Nerelimomab (10 mg/kg)-treated groups. The capsaicin + Nerelimomab group received Nerelimomab (10 mg/kg) 1 h before capsaicin treatment. Based on the weight of the C57BL/6N mouse, the concentration of capsaicin solution was adjusted to ensure the quantity of capsaicin administered was 30 or 40 mg/kg. All treatments were administered to the lungs via a nebulizer. Survival was monitored for several days post-treatment. Based on the survival curve and changes in IL6 and IL18 in lung tissue, the concentrations of capsaicin and Nerelimomab were selected for in vivo experiments. Other experiments were conducted according to the treatment diagram shown in Figure 6, and the survival rate was obtained by observing the mice for 7 days. On the third day after capsaicin administration, the mice were euthanized, and lung tissue was collected for subsequent experiments. In the acute lung injury model, the animals showed obvious symptoms after 3 days of modeling, which was a common time point for biochemical index detection. The time of death following acute lung injury caused by different chemicals and doses is different. Longer observation of the survival curve would assist in better understanding of the therapeutic effect of the investigated drug.

### 4.3. Cell Culture and Treatment

A549 cells (American Type Culture Collection [ATCC], Manissas City, VA, USA) were cultured in McCoy’s 5A medium (Gibco, New York, NY, USA) supplemented with 10% fetal bovine serum (FBS; Gibco) and 1% penicillin/streptomycin (Gibco) in an incubator set at 37 °C, containing 5% CO_2_, and 95% humidity.

Next, 2 mL of A549 cells (5 × 10^4^ cells·mL^−1^) were seeded in six-well plates (Cellvis, Mountain View, CA, USA) for 24 h. The cells were then divided into four groups (n = 3 for each group): control; Nerelimomab control (30 μM Nerelimomab); capsaicin (300 μM capsaicin); and capsaicin + Nerelimomab (300 μM capsaicin and 30 μM Nerelimomab) groups. All experiments were conducted in triplicate with different batches of cells.

Cell viability was measured using a CCK-8 assay kit (Dongren Chemical Technology Co., Ltd.). Briefly, A549 cells (100 μL, 5 × 10^4^ cells·mL^−1^) were seeded in 96-well plates for 24 h. The cells were then treated with serial concentrations of capsaicin for 24 h. Co-treatment of cells with 30, 40, or 50 μM Nerelimomab and 300 μM capsaicin for 24 h was used for the inhibitor group. All assays were performed according to the manufacturer’s instructions [38]. Based on the CCK8 results and the changes in IL6 and IL18 levels in A549 cells, the concentration of 30 and 300 µM were selected for capsaicin and Nerelimomab, respectively, in the in vitro experiments.

### 4.4. Transcriptome Testing and Bioinformatics Analysis

Lung tissue samples were used for transcriptome testing. Total RNA was extracted using TRIzol reagent (Invitrogen, New York, NY, USA), following the manufacturer’s instructions. RNA quality was then assessed using an Agilent 2100 BioAnalyzer (Agilent Technologies, Santa Clara, CA, USA) and a Qubit Fluorometer (Invitrogen). Samples with an RNA integrity number > 7.0 and 28S: 18S ratio > 1.8 were used in subsequent experiments. RNA-seq libraries were generated and sequenced by Capital Bio Technology (Beijing, China). Additional methodological details can be found in our previous study [38]. Statistically significant differentially expressed genes (DEGs) were identified by log fold change (log FC) values ≥ 1 and adjusted *p*-values ≤ 0.05. Statistical significance was set at *p* < 0.05.

### 4.5. Hematoxylin and Eosin (HE) Staining of Lung Tissue Sections

Mice were anesthetized, and the lower lobe of the left lung was collected, fixed at 4 °C in 4% formaldehyde in phosphate buffered saline (PBS) for 24 h. The tissue samples were processed, embedded in paraffin, and sectioned into 4 μm sections using a microtome. Subsequently, sections were deparaffinized using xylene, rehydrated through a descending ethanol series, and rinsed with distilled water. HE staining of lung tissue samples was then performed [39]. Dehydration was performed using ascending ethanol concentrations and clearing was conducted using xylene. Finally, lung sections were visualized under an inverted microscope (IX71; Olympus, Tokyo, Japan).

### 4.6. Quantitative Reverse Transcription PCR

Total mRNA was extracted from cells and lung tissue samples using TRIzol reagent (Invitrogen). cDNA was prepared from 1 μg of total mRNA using a TaKaRa Reverse Transcription Kit (G469851237; TaKaRa, Osaka, Japan). cDNA samples were amplified with real-time PCR using the Universal SYBR Select Master Mix in a StepOne real-time PCR system (Applied Biosystems, Carlsbad, CA, USA). Primer Premier 5.0 software (Premier Corporation, Ottawa, ON, Canada) was used to design primers. The primers used in this study are listed in Table 1 for mouse lung tissue and Table 2 for A549 cells. Four technical replicates were performed for each sample and actin was used as a housekeeping gene.

### 4.7. Protein Detection by ELISA

A549 cells (1 × 10^7^ cells) or lung tissue (50 mg) were washed thrice with PBS. Following this, PBS (1 mL) was added to the lung tissue or cells and the mixture was ground thoroughly. The supernatant was obtained by centrifugation. Next, a 96-well plate was placed at room temperature to equilibrate for 20 min prior to adding 50 µL of different concentrations of standard substances and sample to each well. Horseradish peroxidase-labeled antibody (100 µL) was added, and sealed reaction wells were placed in an incubator set at 37 °C for 60 min. The wells were washed 5 times with washing solution, luminescent substrate was added, and the mixture incubated in the dark for 15 min. The OD was determined at 450 nm following addition of the terminating solution. More details regarding these procedures can be found in the manuals of Bcl-2 ELISA kits (EY-01H1851, Shanghai Yiyan Biological Co., Ltd., Shanghai, China), Bax ELISA kits (BLL108422E, Shanghai Baililai Biological Co., Ltd., Shanghai, China), and Cleaver caspase-3 ELISA kits (YT-M32210, Tianjin Yueteng Biotechnology Co., Ltd., Tianjin, China).

### 4.8. Apoptosis Detection by Flow Cytometry

A549 cells were digested using 25% trypsin at 37 °C for 3 min. Cells were then collected in centrifuge tubes using a cell scraper. After terminating digestion, the samples were centrifuged at 1000 rpm for 5 min and washed thrice with PBS. The cells were resuspended in 1000 μL of Annexin V binding buffer. Subsequently, Annexin V-FITC (5 μL) and propidium iodide (PI; 5 μL) were added and the mixture incubated in the dark for 15 min. Detection was performed using a BD FACSCalibur Flow Cytometer (Becton, Dickinson and Company, Franklin Lake, NJ, USA) [40].

### 4.9. Apoptosis Detection by Confocal Imaging

A549 cells were washed three times with PBS. Subsequently, Annexin V-FITC (1/1000, *v*/*v*), PI (1/2000, *v*/*v*), and Hochest33342 (1/2000, *v*/*v*) were added and the mixture was incubated in the dark for 15 min. Image capturing was performed using a Keyence BZ-X800LE confocal system (Keynes (China) Co., Ltd., Shanghai, China).

### 4.10. Statistical Analysis

Data are expressed as mean ± standard deviation (SD). Statistical significance was determined using Student’s *t*-test; where a *p*-value < 0.05 was considered statistically significant. Statistical tests were performed using (Graphpad Prism 9 software (Graphpad software Company, San Diego, CA, USA)).

## 5. Conclusions

This study demonstrates that capsaicin inhalation can contribute to ALI. Based on transcriptome and proteome analysis, our findings highlight that capsaicin exposure leads to the release of pro-inflammatory factors, particularly TNF-α, which initiates a cascade of events that result in apoptosis and exacerbation of lung injury. The use of Nerelimomab, a TNF-α antibody, mitigates these effects, reducing inflammatory cytokine levels and improving survival outcomes in C57BL/6N mice. These results emphasize the importance of regulating the TNF signaling pathway as a therapeutic strategy to alleviate capsaicin-induced lung damage. Overall, this research provides an in-depth understanding of the taxological mechanisms of capsaicin. Future studies should focus on optimizing the clinical application of Nerelimomab (assessment of its bioavailability, pharmacokinetics, and safety) to enhance safety and mitigate the adverse effects of capsaicin exposure. Further, more applications of Nerelimomab in clinical treatment should be explored.

## Figures and Tables

**Table 1:** Primers for C57BL/6N lung tissue.

Gene Name	Forward Primer	Reverse Primer	Amplicon Size
IL-6	CTGCAAGAGACTTCCATCCAG	AGTGGTATAGACAGGTCTGTTGG	131
IL-18	GTGAACCCCAGACCAGACTG	CCTGGAACACGTTTCTGAAAGA	202
TNF-α	CTGAACTTCGGGGTGATCGG	GGCTTGTCACTCGAATTTTGAGA	122
Bcl-2	GCTACCGTCGTGACTTCGC	CCCCACCGAACTCAAAGAAGG	147
BAX	CCGGCGAATTGGAGATGAACT	CCAGCCCATGATGGTTCTGAT	229
Caspase 3	CTCGCTATAGTACGGATGTG	TCCCATAAAGGACCCCTTCATCG	201
Actin	CCCAAAGCTAACCGGGAGAAG	CCAGAATCCAACACGATGCC	144

**Table 2:** Primer for A549 cells.

Gene Name	Forward Primer	Reverse Primer	Amplicon Size
IL-6	ACTCACCTCTTCAGAACGAATTG	CCATCTTTGGAAGGTTCAGGTTG	149
IL-18	TCTTCATTGACCAAGGAAATCGG	TCCGGGGTGCATTATCTCTAC	75
TNF-α	CCTCTCTCTAATCAGCCCTCTG	GAGGACCTGGGAGTAGATGAG	220
Bcl-2	GGTGGGGTCATGTGTGTGG	CGGTTCAGGTACTCAGTCATCC	147
BAX	TGAAGACAGGGGCCTTTTTG	AATTCGCCGGAGACACTCG	140
Caspase 3	AGAGGGGATCGTTGTAGAAGTC	ACAGTCCAGTTCTGTACCACG	81
Actin	TGCCAACAACGTCATGTCG	CAGCGCGGTGATCTCTTTCT	107

## Data Availability

Data are contained within the article and Supplementary Materials.

## Supplementary Materials

The following supporting information can be downloaded at: https://www.mdpi.com/article/10.3390/ph17121694/s1, Figure S1. Molecular structure of Nerelimomab; Figure S2. TNF-α mRNA expression, Bcl-2/Bax mRNA ratio, and Caspase 3 mRNA expression in the lung tissue of C57BL/6N mice (n = 6); Data are represented as mean ± SD; Figure S3. TNF-α mRNA expression, Bcl-2/Bax mRNA ratio, and Caspase 3 mRNA expression in A549 cells (n = 3); Data are represented as mean ± SD; Figure S4. (A) The compensation for Figure 4E (B) The gating strategy for Figure 4E; Figure S5. The expression of TRPV1 mRNA in capsaicin-induced acute lung injury, n=6; Data are represented as mean ± SD.
