# Supplementary material for: Nerelimomab Alleviates Capsaicin-Induced Acute Lung Injury by Inhibiting TNF Signaling and Apoptosis
Source: Pharmaceuticals (Basel). 2024 Dec 15;17(12):1694. doi: 10.3390/ph17121694 (PMC11676931; doi:10.3390/ph17121694)
Supplement: Supplementary file 1 [file pharmaceuticals-17-01694-s001.zip › pharmaceuticals-3273963-supplementary.pdf]

Nerelimomab alleviates capsaicin-induced acute lung injury by inhibiting TNF signaling and apoptosis

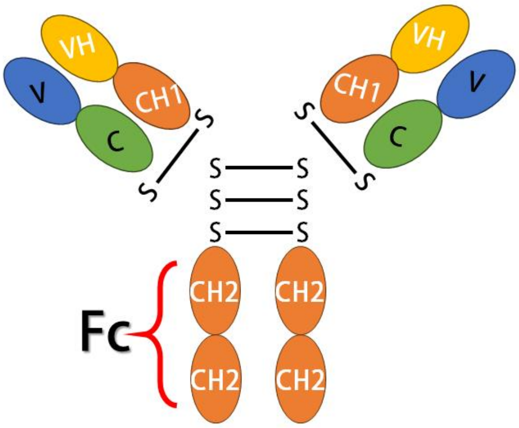

Figure S1. Molecular structure of Nerelimomab

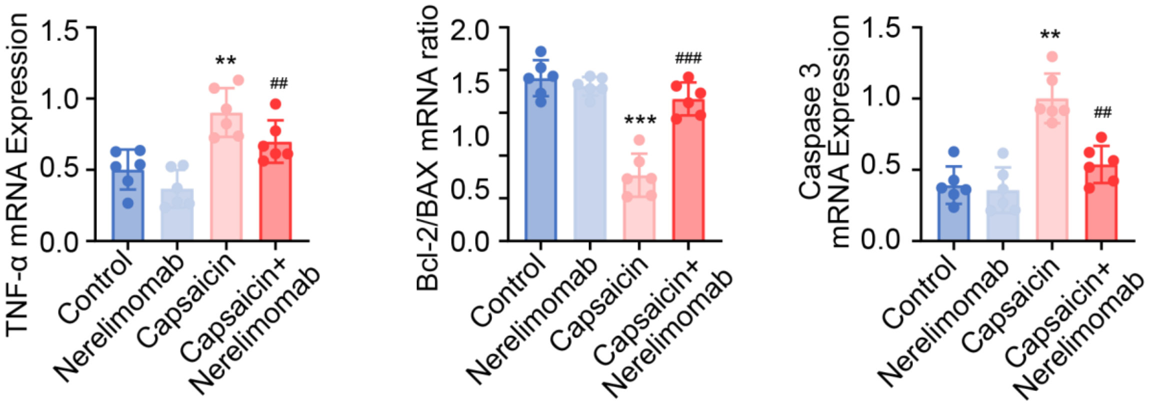

Figure S2. TNF-α mRNA expression, Bcl-2/Bax mRNA ratio, and Caspase 3 mRNA expression in the lung tissue of C57BL/6N mice (n=6); Data are represented as mean ± SD; ** $p < 0.01$ and *** $p < 0.001$ vs. the control group. Data are represented as mean ± SD; ## $p < 0.01$ and ### $p < 0.001$ vs. the capsaicin group.

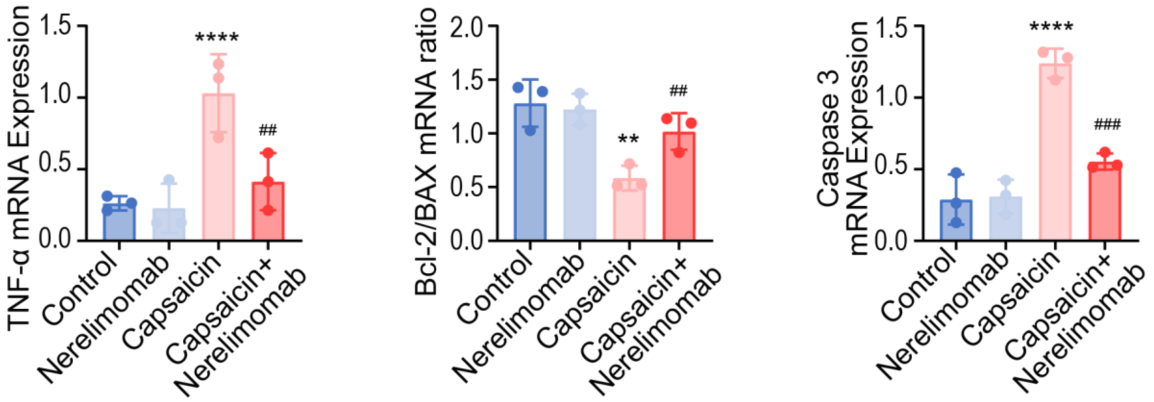

Figure S3. TNF-α mRNA expression, Bcl-2/Bax mRNA ratio, and Caspase 3 mRNA expression in A549 cells ($n = 3$); Data are represented as mean ± SD; ** $p < 0.01$ and **** $p < 0.0001$vs. the control group. Data are rep-resented as mean ± SD; $^{\#}$ $p < 0.05$, $^{\#\#}$ $p < 0.01$ and $^{\#\#\#}$ $p < 0.001$ vs. the capsaicin group.

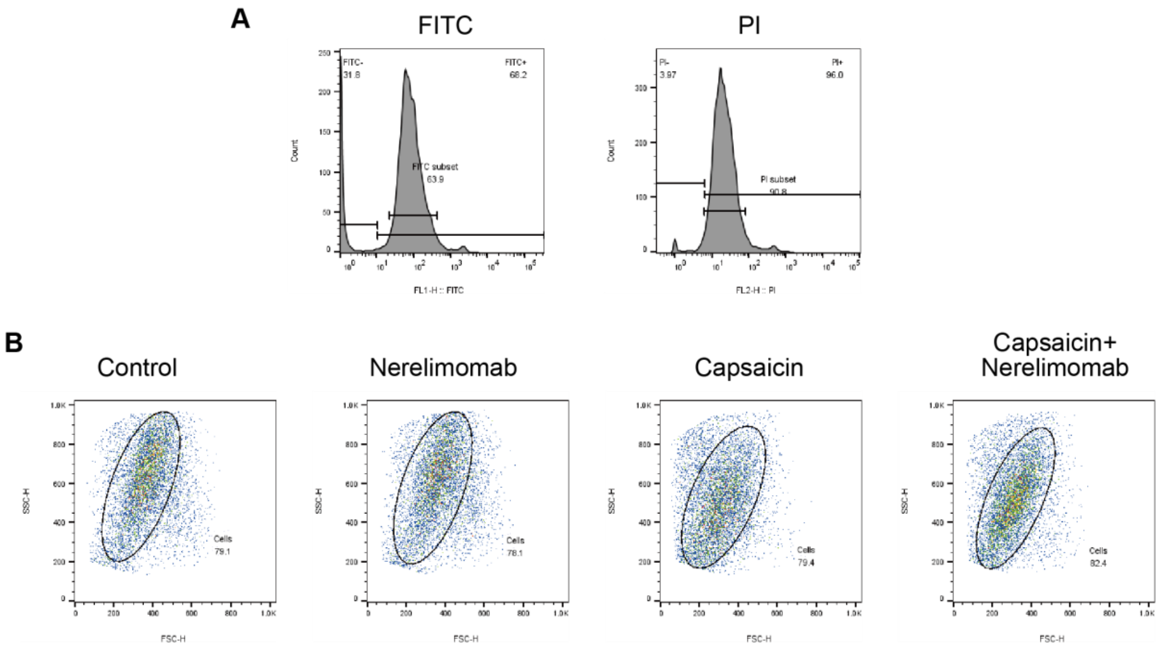

Figure S4. (A) The compensation for Figure 5E
(B) The gating strategy for Figure 5E.

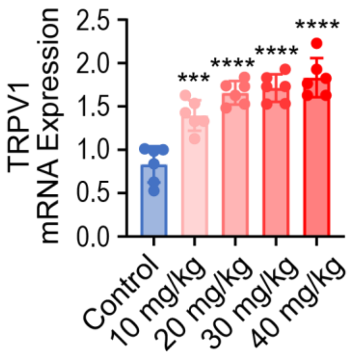

Figure S5. The expression of TRPV1 mRNA in capsaicin-induced acute lung injury, n=6; Data are represented as mean ± SD; *** $p < 0.001$ and **** $p < 0.0001$ vs. the control group.
